# Revisiting the Digital Plumber: Modifying the Installation Process of an Established Commercial IoT Alarm System

**DOI:** 10.1007/s10606-022-09455-2

**Published:** 2023-03-04

**Authors:** Teresa Castle-Green, Stuart Reeves, Joel E. Fischer, Boriana Koleva

**Affiliations:** Mixed Reality Lab, University of Nottingham, Nottingham, UK

**Keywords:** Design practice, Internet of things, Industry, Work practice Infrastructuring, Digital plumbing, Infrastructural inversion, Ethnographic study, IoT alarm

## Abstract

The ‘digital plumber’ is a conceptualisation in ubicomp research that describes the work of installing and maintaining IoT devices. But an important and often understated element of *commercial* IoT solutions is their long-term socio-technical infrastructural nature, and therefore long-term installation and maintenance needs. This adds complexity to both the practice of digital plumbing and to the work of design that supports it. In this paper we study a commercial company producing and installing IoT alarm systems. We examine video recordings that capture how a digital plumbing representative and software development team members make changes to both the installation process and supporting technology. Our data enables us to critically reflect on concepts of infrastructuring, and uncover the ways in which the team methodically foreground hidden elements of the infrastructure to address a point of failure experienced during field trials of a new version of their product. The contributions from this paper are twofold. Firstly, our findings build on previous examples of infrastructuring in practice by demonstrating the use of notions of elemental states to support design reasoning through the continual foregrounding and assessment of tensions identified as key factors at the point of failure. Secondly, we build on current notions of digital plumbing work. We argue that additional responsibilities of ‘reporting failure’ and ‘facilitation of change’ are part of the professional digital plumbing role and that commercial teams should support these additional responsibilities through collaborative troubleshooting and design sessions alongside solid communication channels with related stakeholders within the product team.

## Introduction

An understudied element of the Internet of Things (IoT) is its long-term socio-technical infrastructural nature and the impact this has on the work of designers. While, of course, IoT solutions have been considered, within Ubicomp literature as a networked infrastructure of physical devices linked through software (for example, Da Costa et al. (2008)), this research does not consider the wider socio-technical views of the infrastructures. The view of IoT solutions as socio-technical infrastructures fits closely with some of the underlying concepts in CSCW. It has thus been suggested that concepts such as; coordinative mechanism and artifacts, local contexts and awareness may help to inform design work relating to IoT technologies and that in-depth studies of IoT applications in use are needed to further explore the relations between humans and technology in this domain (Robertson and Wagner, 2015). A further topic recently discussed in detail within CSCW which is particularly relevant to the ongoing work of designers within the field of IoT is that of ‘infrastructuring and collaborative design’ (Karasti et al. 2018). This perspective considers the design and evolving nature of socio-technical information infrastructures as a topic of interest to CSCW researchers.

Information infrastructures as described by Neumann and Star (1996) are relational in nature; incorporating human, social and technological elements to make up a system as a whole. Key features of information infrastructures, which present very specific challenges for designers and developers working with them, are that of embeddedness into structures and arrangements (some of which are social processes), and invisibility-in-use (Star and Bowker, 2006). While a number of studies in the ubiquitous computing literature have touched on this social embeddedness (for example, Tolmie et al. (2002)) they focus on embedding ‘things’ into social arrangements, and the implications this has for design and the end-user, rather than considering the socio-technical layers within the product and service itself. In contrast, a number of CSCW studies in this area have focussed on the wider perspectives of infrastructuring, often considering the social and political engagement aspects of large scale public infrastructures (such as, civic engagement (Korn and Voida, 2015), healthcare systems (Simonsen et al. 2020) and community networks (Crabu and Magaudda, 2018)). Practice-based studies, such as the one described in this paper, are required in order to gain a focussed view of the socio-technical infrastructural relations within IoT solutions themselves and the impact that this has on the design and management of commercial IoT products and services. In particular, unpacking the ways in which infrastructuring work naturally unfolds within this type of commercial environment.

A further, related, consideration for the design of IoT solutions is that of their ongoing service nature, a particularly prominent feature in commercially deployed solutions. With this comes the need for management and maintenance work similar to that described by Orr (1996) in relation to photocopiers. This type of installation and maintenance work has been explored to some extent within ubiquitous computing and CSCW, mainly centered around smart homes. For example, discussions around the work of ‘digital plumbers’ undertaking research deployments into real homes (Tolmie et al. 2010) and householders engaging in home networking (Grinter et al. 2009). Building on the work of Bowker (1994), these papers demonstrate the role of digital plumbing as a core element in establishing IoT systems and as such, highlight a need for systems to support the social order and evolving routines of people installing and maintaining them. More recently, research on the role of digital plumbers has examined the installation of IoT solutions into the industrial setting of a production company (Castelli et al. 2022). In line with much of the work in this area, this study focused on the initial installation of a system and highlighted challenges relating to coordination of multiple parties and job roles within the installation work of an energy management solution in a large business. While these papers highlight important aspects of digital plumbing work they leave unanswered questions in relation to the longer-term infrastructural work required by professional digital plumbers^1^ alongside software engineers and designers, to manage and modify these solutions over time.

Fox et al. (2018) focused on the wider impact of IoT deployments by highlighting the roles and concerns of organisational actors with collective responsibility for maintenance. In particular, they demonstrate a need to consider longer-term elements of IoT installations in relation to use, service and reworking of infrastructures. Odom et al. (2016) suggestion of the ‘research product’ further supports this growing call to address questions of embeddedness and interaction beyond that of the research prototype and initial installation work. While these papers build nicely on the call to ‘move beyond the lab’ (Fox et al. 2006), practice based design studies are also required to deepen our understanding of the various complexities involved in providing support for professional digital plumbers working within industry settings. Particularly in relation to the collaborative nature of the infrastructuring and design work that supports it.

By taking an ethnographic approach to study the socio-technical work of industry practitioners, we contribute to the practice-oriented program of CSCW (Schmidt, 2009). Our approach is consonant with a broad ‘turn to practice’ in HCI (Kuutti and Bannon, 2014) and a long history of studying infrastructures in CSCW (Karasti and Blomberg, 2018). Through this we aim to increase our understanding of the work of practitioners within the commercial field of IoT, so as to better define related roles and approaches to modification and maintenance of commercial IoT systems. In particular, we unpack a trouble initiated project meeting supporting the work of the ‘professional digital plumber’. In our reporting of this we unpack the ways in which an industry based project team navigate the infrastructuring work of uncovering the relational tensions at the centre of the trouble and exploring a solution to resolve those tensions.

In the following sections we will discuss the conceptual foundations on which this work is based including infrastructuring theory and IoT design challenges. Following this, we provide details of our research setting and approach, before unpacking fragments of data drawn from a project meeting that addressed various installation troubles experienced by field engineers during trials of the updated IoT alarm system. We conclude by describing the ways in which our findings build on the notion of the digital plumber expanding the current view of their role to consider additional responsibilities as part of their professional practice. We also demonstrate the use of infrastructural inversion (Bowker, 1994) as a means to support design reasoning in practice. We build on previous descriptions of infrastructural inversion through the identification and demonstration of the use of notions of elemental states to provide purchase to stakeholders as they collaboratively work towards a design solution.

## Conceptual foundations of infrastructuring and IoT design

CSCW has long had a conceptual and practical interest in notions of infrastructure. Here we review some of this work to situate our study’s concern for infrastructuring, as well as examining the nexus of CSCW concerns for work practices and the ubiquitous computing literature. We will first discuss infrastructuring as an approach to design, including the use of infrastructural inversion to navigate the core challenges of working with information infrastructures. Secondly, we consider previous work in relation to design challenges specific to the context of commercial IoT solutions.

### Infrastructuring as an approach to design

To ground our work we draw on conceptualisations of information infrastructures as socio-technical and relational in nature. Neumann and Star (1996) are credited with introducing the notion of socio-technical information infrastructures to the field of design. Their work reports on the development of a digital library system commissioned by the US government. Drawing on the field of Science and Technology Studies (STS) and previous works (Star and Ruhleder, 1994; Ruhleder and Star, 1996) they highlight the relational nature of infrastructures and the levels of complexity involved in infrastructural work. Through this Neumann and Star present a view of information infrastructures that looks beyond the object view to consider the relationship of infrastructural elements to people and activities. They drew on Bucciarelli’s notion of object worlds as a central concept impacting participatory design, with a focus on bringing together object worlds as object universes. Within his influential works Bucciarelli (1988) suggested, particularly within organisational design settings, participants often come from very different ‘object worlds’, which incorporate the knowledge, tools, processes and cultures associated with their disciplines, departments and job roles. He argued design participants coming from different object worlds approach and see the design differently. It is important to note that for Neumann and Star, infrastructure is situated, i.e. something that happens for someone at a particular point in time. Thus, when considering the socio-technical aspects of design work, the diversity of infrastructural elements and actor backgrounds, presents additional challenges to the complexity of the design task.

Building on Ruhleder and Star (1996), Star and Bowker (2006) specified 8 salient features of infrastructure. These include; embeddedness (in structures arrangements and technologies), transparency (invisibly supporting tasks), reach or scope (beyond a single event or practice), learned about as part of membership (gaining familiarity), links with conventions of practice (shaping and being shaped by practices), embodying standards (plugging-in in a standardised way), built on an installed base (inheriting strengths and limitations) and becoming visible on breakdown (emerging from their invisible state). These salient features provide a sense of the enormity of information infrastructures within the modern connected world and offer a way to help conceptualise and ground our understanding of the related design work or ‘infrastructuring’ involved in working with them. One of the core concepts differentiating infrastructure building from ‘object design’ is the concept of working with the installed base, something Neumann and Star referred to as one of the greatest challenges. Their work suggests concepts of embeddedness within an installed base and invisibility-in-use both pose significant challenges for design work, as by its nature design relies on an element of visibility over its subject. In addition to this, infrastructures need to be able to persist in time, therefore the work of modification is key (Star and Bowker, 2006). Ruhleder and Star (1996) suggest infrastructural inversion as a potential way to address these core challenges.

Infrastructural inversion, as introduced by Bowker (1994) and expanded on by Ruhleder and Star (1996) involves intentionally changing the focus from people and things as the cause of change to also include relations. This change in focus allows the foregrounding of elements usually residing in the background. Thereby attending to such things as the mundane, the routine and unnoticed supporting work that enables the infrastructures to function, such as software algorithms, standards and processes. Infrastructural inversion was traditionally used by researchers looking to uncover infrastructural invisibility for research purposes. More recent views on infrastructural inversion have expanded this notion to include gaining purchase for design from the visibility created in breakdown situations (Pipek and Wulf, 2009) or through incorporating stakeholders for whom infrastructuring is part of their everyday work (Parmiggiani, 2015). While initially this approach was about researchers understanding the nature of the infrastructure, more recently it has been considered as a method used by participants of the setting being studied (Parmiggiani, 2015) and suggested as a generative approach to design (Korn and Voida, 2015; Simonsen et al. 2020).

The work of Pipek and Wulf (2009) draws on infrastructuring as a way to conceptualise organisationally based IT solutions thus focusing on ongoing design challenges within the workplace. They refer to work infrastructures to consider the complexities of these systems and the ways in which physical systems are embedded within social infrastructures. Their framework refers to ‘points of infrastructure’ (moments when the infrastructure becomes visible to the user due to breakdown) which subsequently support a search process leading to design activity or innovation. This results in changes to the work process, technology or both. While the work of Pipek and Wulf is useful for expanding the view of infrastructuring and providing insight into the creative processes that lead to change, their descriptions of the design work itself are fairly general with little detail on how the work is practically accomplished beyond the point of infrastructure—a limitation that Bødker et al. (2017) also alludes to. A particularly interesting point about Pipek and Wulf’s approach, however, is their take on the human element, with actors seen as both a creative resource driving change and an infrastructural element. By this we mean that the knowledge-bases, work practices and related resources relied upon to achieve assigned tasks and goals are all considered as part of the infrastructure. Bowker et al. (2009) also specifies individuals within related roles such as designers, developers and users as being included as elements under the conceptual banner of information infrastructures.

Much of the research on information infrastructures relates to researchers working with information infrastructures such as, online library systems (Neumann and Star, 1996), work infrastructures (Pipek and Wulf, 2009; Simonsen et al. 2020), infrastructures of civic engagement (Korn and Voida, 2015) and oil and gas suppliers (Parmiggiani, 2015). While elements of the internet of things (IoT) feature as parts of these infrastructures (for example, the smart-boards discussed in Simonsen et al. (2020)) in that technology change influences and embeds into social process, there is a lack of research looking at how this features within the IoT industry itself, in particular, *how* infrastructuring as a process supports the work of technology design and development within this domain.

### IoT systems as socio-technical and relational in nature

Due to both the heterogeneous and long-term nature of managed IoT systems, complexities of stakeholder diversity often materialises. These diverse actors may include, hardware engineers, software engineers, technicians, product owners, network specialists, data scientists, user interface (UI) designers, solutions architects and field engineers – this level of diversity is of course not necessarily unique to the field of IoT, however, it is a notable challenge for collaborative design work in this context as there are a number of differing ‘object worlds’ (Bucciarelli, 1988) to navigate and combine during design reasoning activities.

It is notable that many of the design challenges of interest to HCI and CSCW in relation to the IoT or more specifically ubiquitous computing, are in line with those discussed in the infrastructuring discourse. Since Weiser’s vision of ‘invisible’ computing (Weiser, 1991), much of the focus of this area of research has been on the capabilities, opportunities and limitations of the technologies themselves (Gubbi et al. 2013; Patel et al. 2017), regarding the embedding of technologies into the home environment and domestic routines (Crabtree and Rodden, 2004; Soro et al. 2018), use and privacy of data (Fischer et al. 2016; Lodge and Crabtree, 2019) and that of user interactions with domain specific devices (Brereton et al. 2015). The design insights presented within these areas of research all allude to challenges of embeddedness, links to practice, working with installed bases and (in)visibility as core within IoT design settings.

Tolmie et al. (2002) delve into the challenge of invisibility-in-use within domestic settings and discuss ways in which ubiquitous computing can be embedded seamlessly and naturally into users’ routines. They discuss a design focus on augmenting resources available to action as part of routines as a way to achieve this invisibility-in-use and note that care must be taken so as not to disrupt the routine that is being supported. Dourish (2004) builds on this noting the relational aspects between physical infrastructure and activity. His paper suggests context is a central issue for ubiquitous computing design and highlights challenges relating to both its situated and relational nature. These papers further elaborate on the core IoT design challenges of (in)visibility and embeddedness and suggest areas for designers to focus their attention. They do however, alongside much of the ubiquitous computing literature, place their emphasis on the initial design of a product and the related considerations for designers looking to embed a ‘thing’ into the domestic setting.

It has been previously suggested that as embeddedness is core to the vision of ubiquitous computing it is important for research to move beyond the lab into real-world deployed settings (Fox et al. 2006). Building on this call for more real-world research and the challenge of seamlessly embedding ‘things’ into domestic settings, Tolmie et al. (2010) considered the installation of devices within these environments, referring to the mundane work of digital plumbing. In their reporting of researchers deploying a prototype into a real home they shed light on the mundane nature of this work and the need for design work to support it. Grinter et al. (2009) presents the view of this installation and maintenance work as something which is often required of household members. These papers highlight the need for systems to both support the social order and evolving routines of the environment in which they are being embedded as well as those of the person doing the installing.

Another factor to consider here, particularly in relation to commercially designed IoT, is the ongoing service aspects of products which mean additional management and maintenance work is often required. In cases where the solutions are managed – as in the case described in this paper – the work of professional field engineers similar to those discussed by Orr (1996) also becomes part of the system or product offering. Thus, there is a need to consider the concept of digital plumbing (Tolmie et al. 2010) as extending beyond the work of researchers and householders (Grinter et al. 2009), to also include professional engineers. Castelli et al. (2022) has recently begun to examine the work of professional digital plumbers through ethnographically unpacking the installation work of an IoT based energy management system within a large business. They highlight complexity in stakeholder roles and relations as a key focus for research moving forwards. However, as with the other papers discussed here their focus remains on the initial installation work to get the system set up. There is little consideration for the ongoing collaborative work required in maintaining, managing and expanding these solutions within the current notion of the digital plumber’s role as a socio-technical part of an information infrastructure.

While research in the field of ubiquitous computing and the IoT has presented these challenges of embeddedness and invisibility-in-use as central features within IoT design, there has been little focus on *how* IoT design work is materially practiced within industry environments. In particular, methods used by practitioners to address and navigate these core challenges within industry contexts. A further challenge facing commercially based design teams that is often missed within research based prototype studies is that of modifying existing systems that are already deployed, i.e., moving beyond the view of initially embedding a thing to also consider the evolving nature of systems and routines.

Building on the discourse around installation and management of IoT solutions and Infrastructuring, we take an ethnographic approach to understanding the work of practitioners within the commercial field of IoT. In this study we unpack a meeting incorporating a professional digital plumber and commercial development team, as they address installation troubles experienced during field trials. Through this we uncover the ways in which hidden and mundane elements of their work processes and underlying technology are brought to the foreground as they work collaboratively to understand the cause of the difficulty experienced by the onsite digital plumber and subsequently design a solution.

## Setting the scene: the problem of unexplained wait times during installation work

The business setting we studied is complex and requires a fair amount of description to make sense of the fieldwork data we present. In short, we performed an ethnographic study of infrastructuring and digital plumbing at work in an industrial setting. The importance of this emerged during our observations of the team trying to deal with the problem of infrastructural breakdown that they noticed happening during the device installation process. Here we also provide details of our stance, and analytic approach to the data collection and analysis.

### Introducing WISP and alert

Our study took place within an SME that has been providing wireless technology solutions for over 30 years. The company (which we will refer to as WISP) is well established in the UK IoT industry with 2 core wireless solutions making up the majority of its portfolio. Within this paper we focus on one of these products in more detail. The solution (referred to hereafter as Alert) provides property and occupancy related data to facilities management companies. The particular sector in question relates to the management of varied occupancy properties, such as holiday lets—properties that need continual oversight by their owners. While there are a number of optional modules that make up the full Alert solution, the main focus within this paper is Alert’s burglar alarm module installed across holiday lets as part of site infrastructure. Importantly, Alert is not a consumer technology, but is sold as a business to business (B2B) managed service. To this end, WISP acts as a service company too, providing the devices and infrastructure themselves as a service which is installed and maintained by their in-house engineers. It is worth noting that this type of business model is becoming increasingly common within the IoT industry, where the ‘ownership’ of the devices remains with the supplier and service contracts are used to run and maintain them. Once again we see here the resonances with Orr’s study of Xerox photocopier technicians (Orr, 1996). Specifically, WISP has a fleet of engineers that install and maintain the entire networked solution, which comprises a Base-station (CIE), Relays (SPT), HUBs and Sensors. Thus the engineers and their work practices can be considered as a core element of the socio-technical information infrastructure that comprises the Alert solution. We also want to reiterate that Alert is a well-established, mature product that has been operating for more than 3 years and is currently deployed in numerous locations across the UK.

**Fig. 1:**
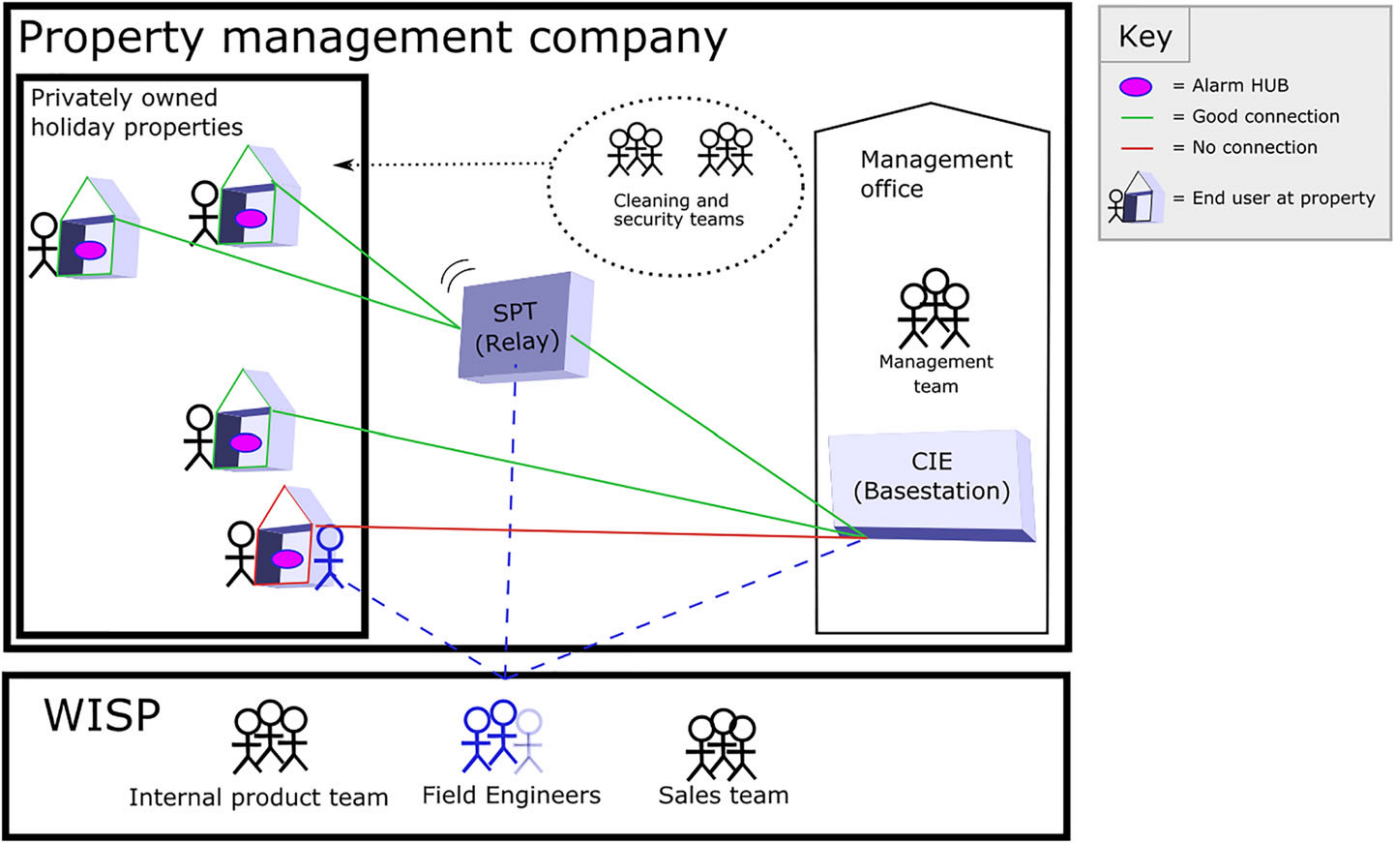
Alert deployment within a network of managed holiday properties

Figure 1 shows an overview of the deployed system, demonstrating the location of the HUBs (individual alarms), SPT (relays to extend signal) and CIE (On-site Base-station linking the network together). It also shows the human elements of the system including employees of WISP, the customer (property management company) and the property owners or guests (end users). It’s worth noting here that the individual alarms are connected to the on-site Base-station via a radio network with the whole holiday home site connected to a particular channel. An additional channel also exists in the form of a wake-up channel which allows devices to be pinged at specific intervals for monitoring and maintenance purposes.

### Project rock: updating the functionality of a live managed IoT system

**Fig. 2:**
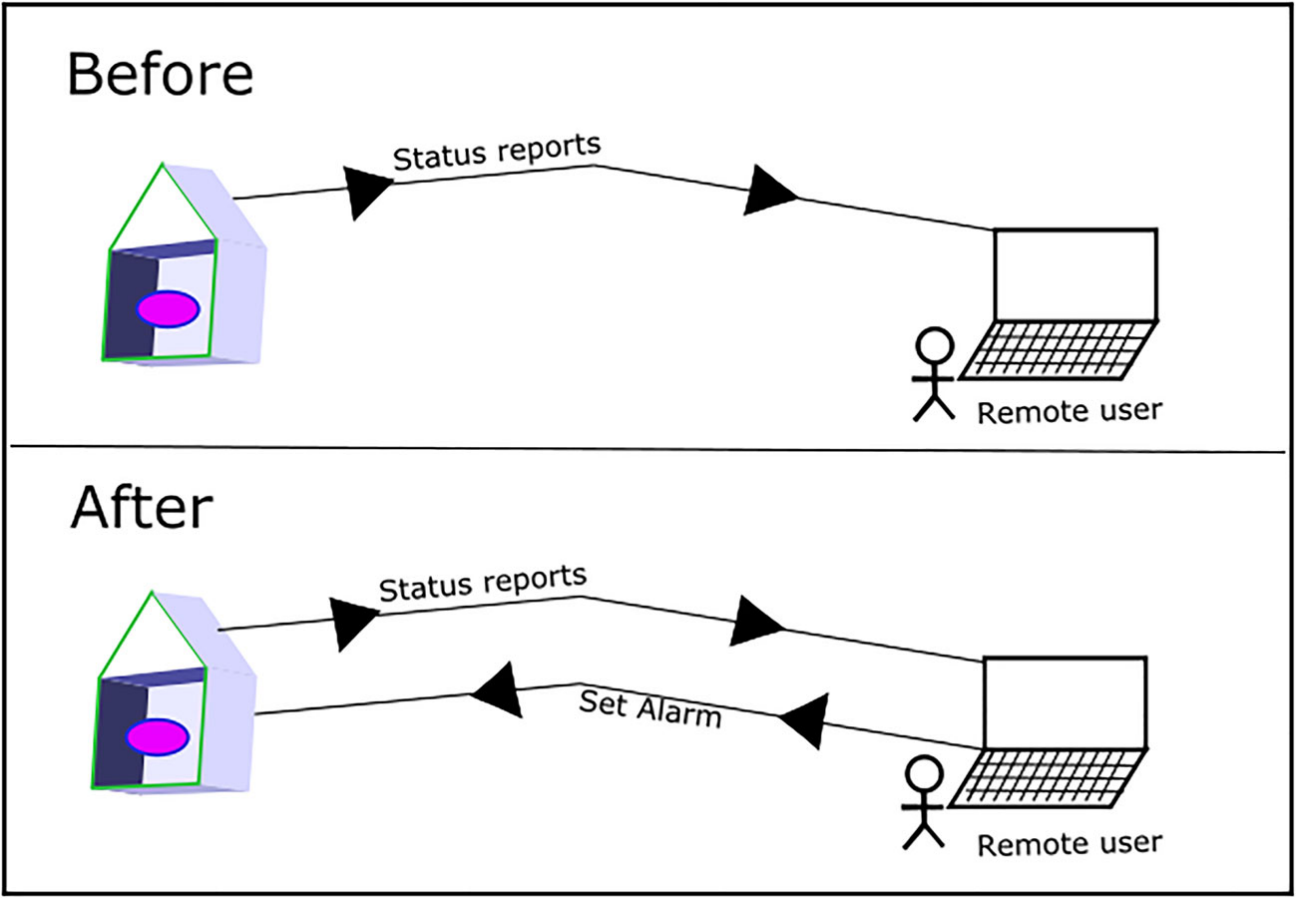
Project rock: additional functionality allows remote alarm setting by property owners

Recent innovation from the product management team at WISP, in relation to improving the Alert product for their customers, led to a project (which we will refer to as Project Rock) involving updating the functionality to allow bidirectional communication^2^. By this we mean that the setting of on-site alarms would be possible remotely, i.e. if the owners or occupants of a holiday let had *not* set the alarm when leaving the property, arming the alarm could instead be done retrospectively via a cloud-based admin system without the need to physically revisit (as shown in Fig. 2).

In order to test the updated functionality before full release, WISP’s project team had arranged, what they referred to as, field trials at 6 customer sites. The field trials involved updating the relevant software versions on a limited number of customer sites who had agreed to take part in the trials. These sites were then closely monitored by the developers, sales team, field engineers and testing teams within WISP to ensure any unforeseen problems could be picked up and dealt with quickly and efficiently. If all problems were deemed to have been resolved within the allocated period the new versions could be rolled out to all customers. However, if problems were ongoing at the end of the allocated field trial time the WISP project team had the option of removing some elements until a later date or extending the trial. The field trial approach was a fairly standard practice adopted by WISP employees as a way of gaining better visibility and testing of any new functionality in-situ before rolling it out across their wider customer bases.

The data in this paper relates to the latter parts of this ongoing cross departmental project. Specifically, the study reported here focusses on a project meeting within the remaining 2 weeks of the development side of the project, where field trials of the new functionality were well underway and for the most part had proven successful. However, some recent trouble had arisen in relation to new installations of alarm systems within the field trial sites. Within this type of facilities management the number of managed dwellings regularly increases, meaning additional installation work within deployed networks is often required—i.e., more alarms need to be put in new dwellings, thus expanding the infrastructure. It is also the case that some maintenance tasks involve an element of installation work, for example, re-establishing connection to an alarm unit following battery replacement. It is for this reason that the practice of digital plumbing is considered a going concern within managed IoT solutions of this nature.

The specific challenge unpacked in this paper was raised by installation engineers (‘digital plumbers’) during the field trial process. In essence, the engineers were experiencing errors and inconsistent wait times as the device attempted to automatically find and connect to the correct network channel. This was increasing the amount of time to complete installation work, thus adding cost to the maintenance side of the business; something which if not carefully managed calls into question the financial viability of the service itself. We report on how the team collaboratively worked towards a solution of enabling the engineers to manually set the correct channel using an RFID tag as part of their installation process. It was thought that this possibility of manual intervention would smooth over these infrastructural issues.

The troubles experienced during installation were initially discussed informally between the Technical Operations Manager (Tom), the Product Portfolio Manager (Phil), and the Test Verification Engineer (Luke) as part of their role of overseeing the field trials. However, due to a lack of clarity over the exact cause of the trouble (as either a software bug or a knock-on effect of a design change), coupled with an urgent need to find a working solution (before the allocated project development time ran out), a project meeting was called with the wider team. Interestingly, topics that may perhaps traditionally be thought of as central to design work—such as core alarm functionality and interactions with end users—were necessarily ‘filtered out’ as the team focussed in on the trouble. They therefore *do not* feature explicitly in any of the discussions, although they are of course present as ongoing concerns.

### Approach to fieldwork with WISP

We selected WISP for fieldwork due to their established nature and focus on B2B managed IoT solutions. They were initially approached by the research team through their website and a series of meetings to discuss the research and establish relationships with the Managing Director, Technical Director and Research and Development Manager followed. A confidentiality agreement was put in place. This arrangement ensured the ethnographer had access to the site to observe work practices including confidential business processes and systems, although of course ultimately WISP decided what access was granted to documentation and what was permitted to be recorded. It also meant that the various approaches taken to the work were chosen and controlled by employees of the company, and care was taken to ensure these were not unduly influenced by the research team—although we recognise that any investigation is necessarily bound up in the production of its phenomena. WISP were also given the opportunity to review research outputs before dissemination to ensure accuracy and confidentiality was maintained. Apart from some limited access to company systems and project documentation, very little restriction was placed on the research.

While this paper focusses on unpacking an in-depth project meeting, it is situated within a wider research project. The research itself took place over a 7 month period which involved; 4 initial meetings with senior staff members, an element of product training through discussions with staff and reviewing printed information, 11 days of onsite fieldwork, interview data (from 3 formal and 4 informal interviews) and detailed field notes from direct observations of work related activities involving employee interactions. It should be noted that due to the timing of the COVID-19 pandemic some of the latter stages of data collection were carried out remotely. 3 hours of video and 3.6 hours of audio data were collected, alongside photographs and 41 A4 pages of field notes. During the fieldwork, complex relations between the operational and technical perspectives of the team became apparent. Attention was paid to this during data collection through focussed observations. The project meeting which forms the basis of this paper was selected as an exemplar of these complex relations between stakeholders, giving an opportunity to foreground the infrastructuring which is integral to the collaborative work of managing and modifying the Alert product. These elements of the work were further investigated during our analysis through a series of 4 detailed data sessions with multiple researchers. Fragments of focus were selected for each data session through systematic analysis of the collected data in line with best practice outlined by Heath et al. (2010). This involved multiple reviews and cataloguing of the whole corpus, followed by the selection of episodes, from the recorded meeting, which demonstrated the complex relational interactions between the different stakeholder groups. Each episode was then transcribed in detail prior to the data sessions.

Follow-up discussions with 3 key project team members to review our reported descriptions of their work, were carried out after the analysis was completed to ensure our descriptions of the activities were both accurate and suitably confidential, as per the initial agreement. Our analytic orientation is ethnomethodological (Garfinkel, 1967) in that we are concerned for describing how members of the team produce or ‘accomplish’ their work. We are also building on existing bodies of research that use ethnographic approaches—sometimes but certainly not always oriented ethnomethodologically—in understanding work practice as it happens (Szymanski and Whalen, 2011; Rouncefield and Tolmie, 2011; Schmidt, 2009). While data presented here has been anonymised, job titles, gender and roles have not been changed.

The fragments of data we present next focus on the resolution of the key trouble raised by the on-site engineering team (‘digital plumbers’) during the field trial process, i.e. (as described earlier) the problem of alarm devices taking inconsistent periods of time to connect to the network and transmit initial data packets. Next, we talk about how the project team responded to this infrastructural breakdown through collaborative design reasoning, during a key 3 hour project meeting called to address and resolve the issue. Transcriptions from the video recording of the meeting are presented as fragments throughout the section to provide clarity and depth to the accounts provided.

## How the project team located and dealt with infrastructural troubles

**Fig. 3:**
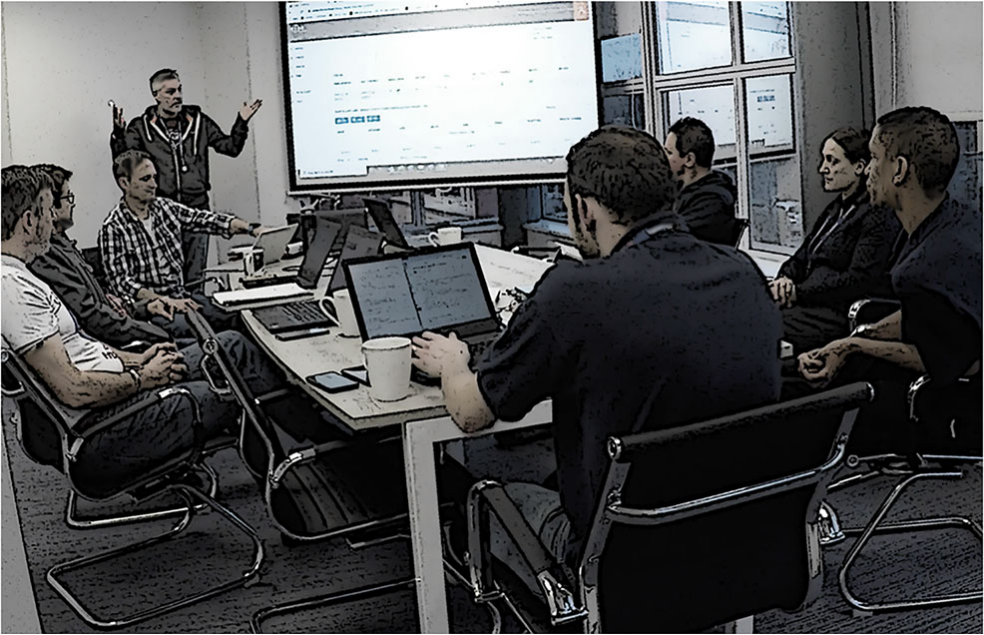
The Project Rock team addressing the installation issues reported during field trials

The WISP team’s problem, of noticing unexplained and inconsistent wait times during Alert alarm installation, were initially communicated informally between the Technical Operations Manager (Tom), the Product Portfolio Manager (Phil), and the Test Verification Engineer (Luke). These initial discussions failed to establish a cause or resolution for the problem and so a more formal approach was taken involving further investigations with the wider project team. The subsequent project meeting (shown in Fig 3) included additional key stakeholders; Software Engineers (Dean and John), R&D Manager (Simon) and Regional Sales Team Leader (Ben). During this meeting Tom was able to formally raise his concerns (as a digital plumbing representative) and initiate the collaborative work of finding a resolution.

**Fig. 4:**
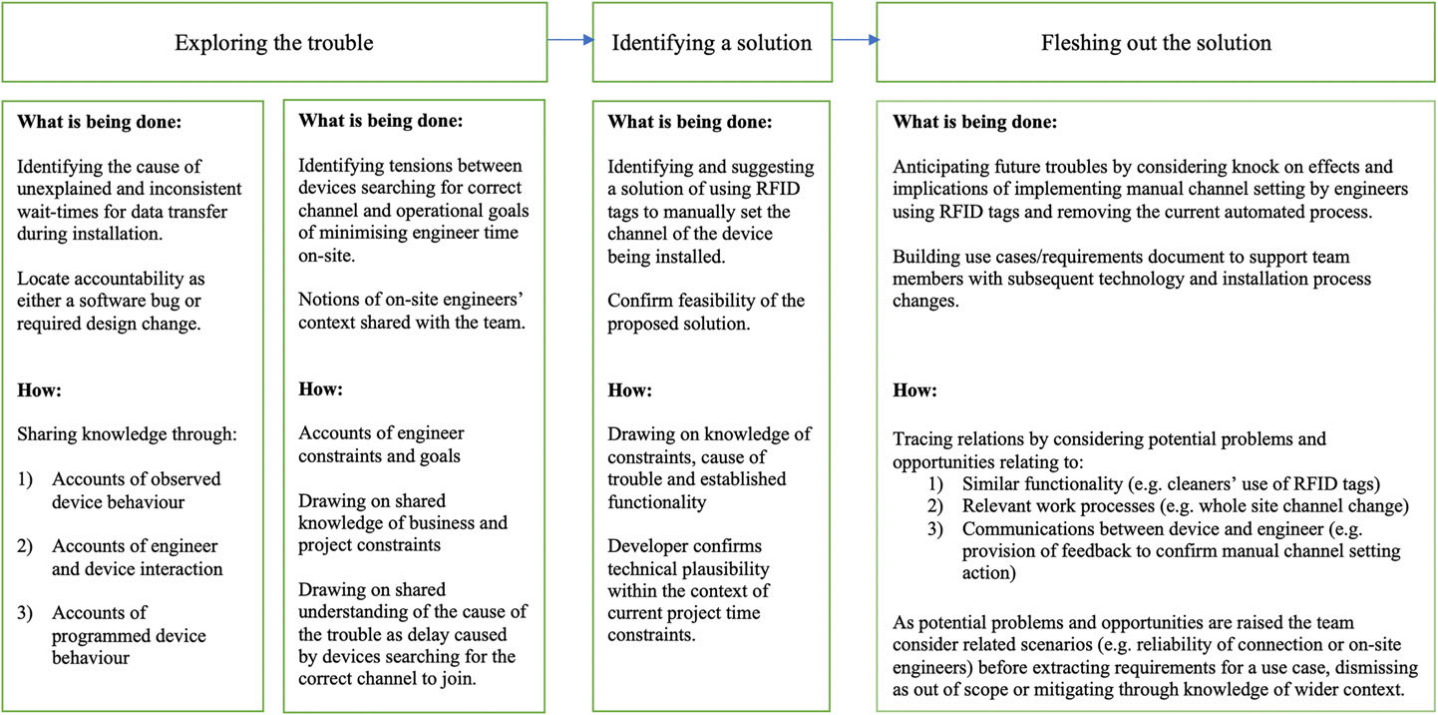
Overview of how the meeting unfolded as the team explore the trouble, identify and then flesh out a solution

As demonstrated in Fig. 4, the meeting itself can be broadly split into three phases. During the first part of the meeting the team’s focus was on understanding the cause of the trouble and deciphering the accountability. For example, a bug in the code would have been accountable to the development team, whereas in the case of a required design change, if a solid case for an urgent change can be presented and accepted, the responsibility of establishing and assessing the impacts of a solution resides with the whole project team.

The second phase of the meeting is much shorter and involves a solution being presented and then confirmed as feasible both from a technology perspective and within the remaining project time-frames. During the final phase of the meeting the focus moves on to fleshing out the solution and anticipating potential future breakdowns. This is achieved through the tracing of related functionality and scenarios. As the team work through this process, queries and suggestions are raised, which are then explored in relation to the connection state and on-site or off-site status of the engineering team. The following sections unpack these phases in further detail.

### Exploring the trouble: identifying relational tensions as the cause of breakdown

To begin with, we look at how members of the team searched for accountability and resolution of the problems at the field trial sites. The team here worked primarily to ‘shape up’ the trouble—to give it some outline, and ‘graspability’. This shaping work occurred during the initial phase of the meeting (i.e. ‘Exploring the trouble’, in Fig. 4) as team members undertook a kind of search process, through reporting and local knowledge sharing of observed and expected behaviours. The following sections explain how the team began this exploration process through the sharing of accounts of device behaviour observed during installation work. This then led to technical accounts of expected or programmed device behaviour within the relevant context of the installation troubles.

#### Exploring the trouble: sharing knowledge through accounts of observed and expected behaviour

The first accounts in the meeting came from Tom (Technical Operations Manager) who reported on difficulties he had personally experienced when undertaking the digital plumbing work of installing new alarm HUBs within the field trial sites. Tom reported troubles during the connection process with occasional error codes, but also, and perhaps more concerningly, inconsistent wait times with no discernible feedback.

Such reporting by Tom served to make the ways in which the underlying infrastructure—normally ‘invisible’—had impeded his work and thus render such problems accountable to the team. While the underlying infrastructural processes (i.e. the software and network connection state) remained invisible to Tom, the lack of feedback, from both the device and cloud monitoring system for an unexpected and inconsistent period of time, became visible in and of itself and as such can be considered as a situated infrastructural breakdown. Through the sharing of a situated account Tom made this ‘point of infrastructure’ (Pipek and Wulf, 2009) accountable to the team as something he considered beyond what could (or indeed *should*) be subject to a work-around by an onsite engineer. Instead, this is something that potentially required *technology change*, whether through the fixing of a bug or through redesign work.

Based on Tom’s reports the team agreed to further investigate, with the focus of the resulting explorations primarily between Tom (as the representative of the onsite engineering team) and Dean (as the lead developer on this area of the project). At this point it remained unclear whether the trouble was the result of a bug in the code, or the result of an unsuccessful design change within the project.

In their continuing exploration of the breakdown context and search for accountability or solution, Tom provided Dean with more detailed accounts of the trouble, incorporating step-by-step descriptions of how the trouble occurred from both engineer and device. Tom used installation and device monitoring logs on the big screen at the front of the meeting to support this description (as shown in Fig. 3) referring to a specific installation that he had been involved in to demonstrate an eight minute delay for installation data to be processed. This additional information served to provide more context to help situate Dean’s technical exploration of the trouble.

Luke also offered some additional information to support Tom’s reports. Luke’s role of Test and Verification Engineer, overseeing the field trials, gave him a different perspective and additional visibility over the device monitoring systems. Within his account Luke reported that when troublesome connectivity behaviour was encountered by Tom during his on-site work, the remote monitoring available to Luke showed the devices *‘wandering off round channels’*.

Notably, this ‘wandering’ behaviour was also reported by Luke as happening at times when devices were trying to rejoin the network after a connection interruption during remote maintenance and not only during engineer installs. Luke’s observation of rejoin behaviour then led to John (Software Engineer) suggesting a simple solution of changing some programmed settings to make the devices *‘more sticky’* on a channel. While this network rejoin issue was resolved quickly and was not the main focus of the meeting it was linked by the team to the core troubles due to similarities in observed behaviour patterns.

These accounts of observed system behaviour shared by Tom and Luke, in the initial phase of the meeting serve to situate the focus of the subsequent discussions on establishing the circumstances surrounding these installation troubles. The result of reports delivered in this way, was the acceptance of the trouble by the wider team as system behaviours which required explanation.

Following these accounts from Tom and Luke, and drawing on internal documentation of the software algorithms, which he requested to be presented on the big screen, alongside his wider knowledge of the technology, Dean was able to identify and explain the context in which this device behaviour would be *expected* from a technical perspective. Within his description he justified the behaviour as a programmed response to a specific type of connection failure where the response message had not been returned from the CIE (Base-station) to the alarm HUB being installed. Dean’s account is shown in Fragment 1. 
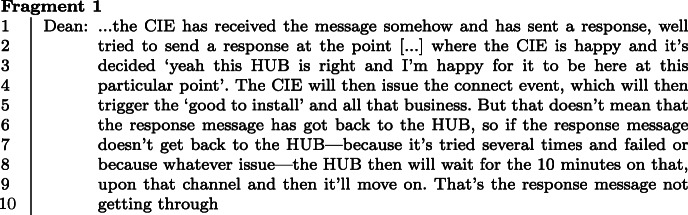


To unpack what Dean is saying, he explains that if the initial attempt to join the network is unsuccessful, devices will make a number of attempts with specific wait times in between and if this continues to be unsuccessful it will conduct a search process of cycling round the channels. Dean’s technical description of the alarm device behaviour and context in which this would occur is designed to provide an explanation for the discrepancies Tom had reported, as well as account for the inconsistency in the time taken for a connection to be established. Dean’s description highlights that the device (as a non-human actant in the connection process) does not have the necessary information about which channel the network it is trying to join, and so when the site is not on the default channel, it is undertaking a channel search process when it doesn’t receive a response from the CIE. Thus, also addressing Luke’s observations of devices *‘wandering off round channels’*.

This explanation of programmed behaviour transforms the problem *away* from being a potential software bug as the cause of the trouble and justifies it as something that has been purposefully designed in response to delays encountered while the device is establishing a network connection. Dean is offering up a design rationale. It is important to note here, the automated connection process, as described by Dean, prioritises operational alarm traffic, rather than installation traffic. It also conserves battery life through the addition of wait times rather than continual processing. These wait times or delays during installation are most likely to be triggered either because the site is not on the default channel, or due to large amounts of operational traffic taking priority at that point in time.

The team has thus by this point in the meeting shaped up observed troubles with the alarm system and also sought to explain them in terms of prior design reasoning. In sum, this led to the team identifying a core issue of inconsistent time-frames for a device to establish its connection during the installation, which simultaneously pointed to possible solutions.

#### Exploring the trouble: identifying and resolving tensions between device behaviour and engineer goals

Working out what was wrong also served to reveal some relational tensions between the programmed behaviour of the device and the needs and expectations of the onsite engineer. The identification of these points of tension, prompted further information to be shared by Tom from his role of Technical Operations Manager. Fragment 2 shows Tom’s response to Dean’s explanation. 
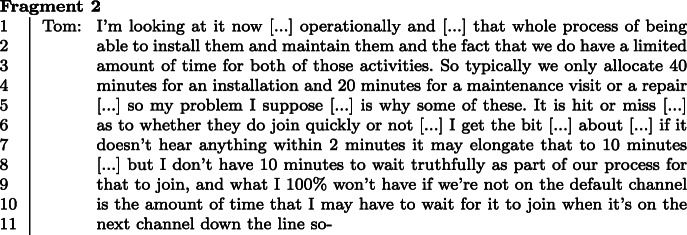


Tom frames his response by reminding others of his operational perspective (line 1). He is Technical Operations Manager, and as such highlights the current solution as conflicting with the goals, constraints and processes of the onsite engineers. Tom highlights time constraints (lines 3-5): while he understands the justification for the devices to wait for a message (lines 7-9), he deems the wait time for a non-default channel as ‘100%’ unacceptable (lines 9-11). This potentially critical comment is softened by his perspectival framing earlier. He further underpins this by positioning himself in the shoes of an engineer with practical on-site constraints to make these points (e.g. ‘I don’t have 10 minutes to wait’), although his management role within the company is to *oversee* this element of the work rather than perform it himself. He is therefore in a position of authority and accountability where the operational elements of the solution are concerned. The additional information Tom provided about the time constraints on engineer site visits serves to foreground the maintenance priorities at a business level and to refocus the task at hand on resolving the tensions between the programmed behaviour of the technology and operational challenges of the installation process.

We see from Tom’s response in Fragment 2 waiting for an ‘invisible’ process^3^ that takes an unpredictable period of time does not fit with goals and constraints of the on-site installation engineer. As part of their shared membership in both the company and the project, the whole project team have an awareness that in order for the solution to be financially viable the maintenance costs to the business need to be carefully managed. While from a practical level, as Tom points out, this means efficient installation times, it also incorporates a need to minimise maintenance visits, which includes battery management. At a business level there is a 5 year battery life target for devices that everyone is continually working towards. This focus on careful battery management ensures a team wide understanding about why devices may need to wait for traffic levels to reduce before reattempting this type of non-critical communication, rather than engaging in a continuous operation.

The above discussions highlight the aligning of goals and values and building of collective knowledge base that formed a core part of these initial exchanges through the foregrounding of infrastructural elements relating to both the work processes of the engineer and the underlying software algorithms. This process enabled the team to identify the exact point of failure *as relational tensions* between programmed device behaviour and the goals and values of the onsite engineer. This is an awareness which realigned the problem space to focus on lack of channel configuration information on the (yet to be connected) device. The result of this additional clarity was that Tom had a fuller picture in terms of the point of infrastructure which thereby enabled the process of user-innovation to successfully take place (Pipek and Wulf, 2009).

### Identifying a solution: suggesting RFID tags as a way to manually set the channel

**Tom’s Solution.** Following the above discussions and drawing on his knowledge that an on-site engineer has access to the channel information that the device needs to complete the operation, Tom was able to put forward a solution. Tom suggested the use of RFID tags to allow engineers to manually set the channel information on devices during installation. This solution capitalised on the engineer’s system knowledge and the capabilities of the devices to receive data from RFID tags (i.e. drawing on opportunities from the installed base (Star and Bowker, 2006)). While not necessarily dealing with the issue of connection failure per se, Tom focused on difficulties experienced when a site is not on the default channel, a point which he indicated as a major tension in Fragment 2 (line 9).

This suggestion of a solution was addressed to Dean as the person with the knowledge and accountability to decipher whether or not it is technically feasible both with the current physical infrastructure and within the remaining project scope and time-frame. Dean’s response was positive thus qualifying the suggestion as valid.

To summarise the above, we have shown how members of the team collaboratively explored and foregrounded *relational elements* between the onsite engineer and the device (as a non-human actant) in the installation process. The result of this led to surfaced tension relating to the time taken for the channel search process conflicting with the on-site engineer’s tight time constraints. Throughout these exploratory discussions the team’s focus remained between two core interacting infrastructural elements at play within both the ‘join’ and ‘rejoin’ processes, i.e. notions of on-siteness and connectedness^4^. These notions were primarily represented by Tom and Dean respectively as accountable to their operational and technical roles.

The meeting did not conclude at this point. With a potential solution on the table, the atmosphere lightened and the team moved on from searching for accountability to design reasoning work which they referred to as ‘fleshing out’ the solution. Changes to the current system would inevitably impact the responsibilities of the engineers, device and development team. For example, through changes in work process or updates to software code. These were further explored in the following discussions.

### Fleshing out the solution: anticipating future troubles by considering opportunities and implications of using RFID tags to set channels

As the team ‘fleshed out’ the solution throughout the remainder of the meeting, they also addressed finer details of the technology and work process changes required for implementation—as well as the potential implications of these. This fleshing out process (as shown in Fig. 4) involved team members taking turns (through self-selection) to raise potential concerns, challenges and opportunities. These raised items derived from the tracing and assessing of links between similar functionality and processes in relation to the two infrastructural notions of connectedness and on-siteness that had been generated in the ‘exploring the trouble’ phase of the meeting. Items were then either; 1) extracted as requirements and added to a document by Simon (as the self-designated note taker for the meeting), 2) dismissed as out of scope or 3) mitigated by foregrounding knowledge of wider context. This process of design reasoning was cyclical in nature with new questions and potential use scenarios raised as others were closed off. It should be noted that while the purpose of this fleshing out was to produce use cases for the development team to work from, this was not a formally organised or facilitated design task. The following sections demonstrate the ways in which the team anticipated future troubles and assessed potential opportunities through this process of relational tracing. We will first discuss the process of considering related functionality as the team consider the connection state of the device and the impact this has on the proposed RFID channel setting process. In the second section we demonstrate the ways in which the team considered related work process both within the installation process and in regards to other maintenance tasks, to assess the impact this design change may have. The final section demonstrates how the focus remained on the initial tensions as the team consider the provision of feedback for the engineer to confirm the RFID based channel setting action has been successfully picked up by the device.

#### Fleshing out the solution: considering related functionality

##### Using RFID Tags Without a Connection

Tom raised initial concerns about whether the RFID tag solution would work prior to the connection between the device and CIE being established. He justified this concern by drawing on similar functionality noting that the RFID tags used by cleaning staff, to unset and reset Alert alarms when accessing holiday properties, were ‘dependant’ on the connection with the CIE. This item was swiftly dismissed by software developer Dean as the code used to process the RFID tags used by the owners (or occupants) of the property to set the alarms, was stored in the HUB, and therefore code was in place to process RFID functionality locally without the need for a connection.

##### Setting the wake-up channel using RFID tags

This approach of exploring links between similar functionality also led the team to consider the opportunity of setting the wake-up channel through the RFID tags as well. As mentioned earlier, there are two channels associated with the devices: 1) the operational channel, which the team are originally addressing with this solution, and 2) the wake-up channel which is separate and serves the purpose of functionally monitoring for maintenance purposes. Fragment 3 shows Dean’s attempted dismissal of this suggestion as he believed it unnecessary due to the wake-up channel being set after a reliable connection between the HUB being installed and the Base-station (CIE) has been established. 
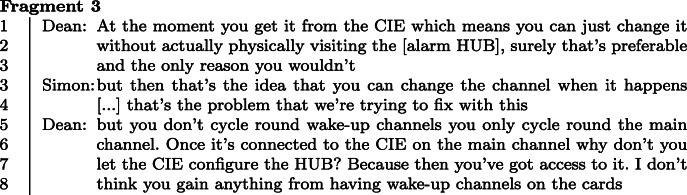


In this example, Dean argues that getting the configuration from the CIE rather than an onsite engineer presenting an RFID tag is a better option. He justifies this by suggesting it prevents the need to physically visit each of the alarm installations (line 2) and that at the point of configuration the connection with the CIE would already be in place (line 6), meaning the device would be in a position to receive that information without the requirement for a search process or human intervention. Simon questioned how related the wake up channel was to the problem at hand (line 3-4), but Dean continued to justify his suggestion that they do not gain anything from setting this by RFID as the channel searching algorithm does not apply to the wake-up channel, it is configured automatically after connection (lines 5-8). Following this conversation Simon then re-framed the need for the RFID solution, suggesting that such RFID setting capabilities act as an additional option to remote setting in this case.

Dean’s initial focus on making changes without ‘physically visiting’ the individual properties presents itself as aligned with the operational business goals of minimising maintenance costs by allowing work to be done remotely where practicable. He further supports this with technical knowledge of the differing functionality between the operational and wake-up channels, with the wake-up channel configuration happening only after the operational connection has been established. This highlights the central nature of notions of connectedness as a resource for design decision making in this case. When Simon questions and finally overrides Dean’s dismissal of the suggestion to include wake-up channel on the RFID tags, he is both showing a distrust of the connection state in terms of time to configure and an understanding that there is likely to be an engineer already onsite at the point of configuration, thus dismissing Dean’s justification of reducing the need to visit the HUB. This further highlights the tensions and complexities between these two infrastructural notions of connectedness and on-siteness and the ways in which the team must balance these as part of their fleshing out work.

#### Fleshing out the solution: considering related maintenance processes

##### Manual whole site channel change

In addition to anticipating potential tensions and breakdowns through considering related functionality, the team also considered the impact of and on related work processes, as part of their work of fleshing out the solution. For example, in Fragment 4 Tom draws on his knowledge of the on-site and off-site maintenance processes to identify additional technology requirements and procedural concerns. This is achieved by considering related maintenance processes that may be impacted by the new solution of engineers setting the operational channel during installation. 
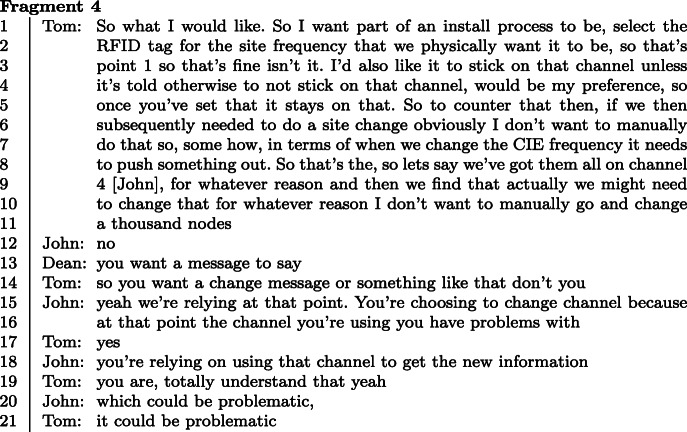


Tom makes his thinking accountable to the rest of the team by talking through a future scenario of an installation using the RFID tag to set the channel (lines 1-3), along with his expectations of the system behaviour to ‘stick on that channel unless it’s told otherwise’ (lines 3-4). He then presents a ‘counter’ scenario in which he would like to be able to change the channel remotely (lines 6-10). Tom raises the concern that in this scenario, where an engineer is not already on-site it would be inefficient to make this change manually, he adds weight by giving an example of ‘a thousand nodes’ (lines 11-12) – a figure that is not unrealistic with this particular product. Dean and Tom then move on to discuss the technology requirements that would be needed to action that change remotely (lines 14-15). At this point, John raises concerns based on the premise that the likely reason for changing the channel a site is on would be in relation to the connection on the channel they’re ‘using’ being unreliable (lines 16-17), it would therefore be potentially ‘problematic’ to action the channel change in this way (line 21). When John uses the term problematic he is referring to the possibility that some devices may not get the channel change message due to an unreliable connection. This could result in Alert alarms losing their network connection, thus rendering them inactive and requiring an engineer visit to reconnect them. Maintaining up-time and functionality for their customers is always a priority for WISP so this type of risk to service would need a level of mitigation. Tom’s acknowledgement on line 21 demonstrates his understanding that a level of on-site readiness and risk mitigation would be needed in this case.

Within this requirements statement Tom drew on the three states of on-siteness that are central to this process, on-site installation and maintenance, off-site maintenance, and minimising the number of required engineer visits. John (in his software engineering role) took the technical perspective of assessing the connection state in relation to the reliability of successful execution of the requested functionality. This tension between the two perspectives further highlights the design complexities related to managing the operational elements of solution maintenance within the limitations of the technology itself.

##### Battery replacement visits

It’s notable the technological and operational accountabilities and perspectives were not limiting in terms of who raised what. For example John raised questions about the process relating to HUB batteries going flat. He suggested that after the battery change, as an engineer would already be on-site, they could use the RFID fob to select the channel again when they reinstall the HUB. While John drew on his own knowledge of the on-site process to make this suggestion, it is notable that he addressed it to Tom as a question, presumably because Tom is the authority in this domain. Tom confirmed John’s suggestion that it could be worked into the maintenance activity as part of the standard work protocol. This exchange also demonstrates how the technology solution and the work processes of the engineers were being designed in parallel within this session.

#### Fleshing out the solution: communications between device and the engineer

##### Device Feedback to Engineers

While up to this point the team had been focusing on the relational aspects of the solution in terms of exploring similar functionality and maintenance processes, there was also a continued focus on the original tension, that of visible knowledge sharing between the device and engineer. As part of this focus, confirmation feedback was an important aspect to be designed. Based on the existing functionality of the devices the team considered both beeping and flashing LEDs as viable options for this to be presented.

During a discussion about the best way to present this feedback to an on-site engineer the team began to consider how many beeps or flashes were needed in relation to the selected channels and whether this should represent both the operational and wake-up channels. In Fragment 5 Tom draws on his knowledge as a member of the on-site engineer team to note that the engineers do not require knowledge of the wake up channel setting at the point of install (line 1). Phil suggested that he (with the assistance of Tom and Ben) would present details of how the interaction with the device should unfold as part of a use case, including the desired behaviour of the device - i.e. entering a mode and providing the user with channel confirmation feedback (lines 3-5). However, John persisted in his exploration, drawing on his knowledge of the internal monitoring systems when he stated that it needed to match the channel number displaying on the ‘cloud’ (lines 6-7) to avoid a ‘disjoint’ (line 11) between the engineers’ knowledge, current confirmation methods and the visual elements of the new RFID based solution. Interestingly Tom’s initial focus was the number that goes on the physical tag to inform the engineers of which channel they are setting it to. While Phil’s comment referred to the interaction and feedback with the device itself, and John’s comment to congruence between the device and the monitoring infrastructure. 
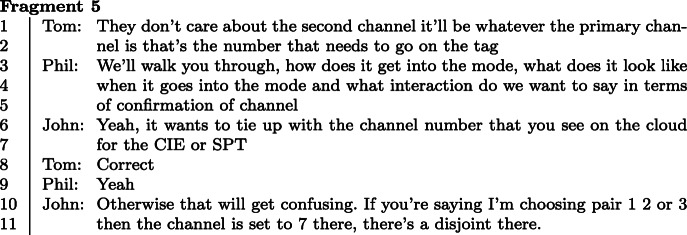


The differing perspectives apparent in this fragment demonstrates some of the complexity involved in these design sessions. It shows how their design reasoning incorporated different forms of interaction between hardware, work processes and supporting software with an overall focus on achieving consistency across the different interaction points. The members make visible their differing accountabilities and perspectives as they collectively map the interaction points from the identification of the correct RFID tag through making the action on the device and receiving feedback from both hardware and cloud based monitoring systems.

Following this design session the solution was successfully implemented within the project time-frames. The only unforeseen challenge that came up for the development team was in relation to another module (i.e. a different device within the same system). As a managed modular system, reuse of code bases is a focus to ensure clarity and overhead reductions in relation to maintenance and updates. An energy monitoring device that makes up part of the modular system did not have RFID functionality built in. This raised the additional challenges of how to deal with an incompatible device. As it was not an option to make hardware changes to comply with the changed functionality it was necessary to silo some of the code-base to ensure the automated channel search was still available in this instance.

## Discussion

In unpacking the work of a commercial IoT project team as they address troubles experienced by field engineers during field trials, we share insights into the way in which infrastructuring is materially achieved in practice. We uncover a number of complexities that are central to the concepts of infrastructuring and ubiquitous computing design, thus shedding light on some of the challenges of managed IoT services. We first focus on the team’s exploration of the breakdown leading to the building of a collective knowledge base and surfacing of related tensions. Secondly, we consider how the team use infrastructural elements identified during the initial exploration as framing to maintain visibility over relational aspects while fleshing out the design. Finally we consider how these insights extend upon the previously documented role of the digital plumber.

### Exploring relational tensions and achieving visibility through infrastructural inversion

In this section we will describe how systematic sharing of reports and accounts were used to give purchase to relational tracing activities, thus giving the team a foundation from which to surface tensions and collectively work towards a resolution. Within our study we see the team investigate a breakdown situation and collectively formulate a workable solution. Firstly they gain purchase for a search process based on an infrastructural breakdown, similar to that described by Pipek and Wulf (2009). Secondly they draw on the local knowledge sharing by key stakeholders and systematic tracing of experienced and expected behaviour to foreground hidden mechanisms and foundational elements of the infrastructure (Star, 1999).

Our findings demonstrate that the smaller field trial management team initially lacked the knowledge and visibility over the software processes to explain breakdowns in connectivity between alarm devices and supporting infrastructure. Therefore other key stakeholders were required as part of an exploration process in order for a resolution to be found. Incorporating key stakeholders in a search for breakdown accountability through knowledge sharing that foregrounds hidden elements and facilitates relational tracing, can be conceptualised as a form of infrastructural inversion (Ruhleder and Star, 1996; Simonsen et al. 2020). We see the project team in our case purposely and systematically foregrounding aspects of a socio-technical system which are routine to them but remain invisible to others.

Pipek and Wulf (2009) refer to meetings between design experts and practice experts as an important infrastructuring activity for supporting new usage innovation. Simonsen et al. (2020) also reported on meetings between super-users working with a participatory design effects-specification approach as an environment in which infrastructuring activities occurred. Based on our findings we also note the importance of meetings between experts to facilitate local knowledge sharing as an activity to foreground related invisible elements and therefore support user-led design. In our case this was a meeting between the operational and technical experts: representatives of the two core perspectives that were present and in tension at the point of breakdown. Neither of these parties were design experts, however, each was an expert in their own domain, namely that of the underlying software, technology capabilities and field engineer work processes. Our study also differs from Simonsen et al. (2020) in that while the participants in our example could be considered as a type of super-user, the task itself was derived from a breakdown situation and was *not* facilitated by the research team. We therefore build on their research by offering a different perspective in relation to how infrastructuring work of gaining visibility of infrastructural elements can be occasioned and self-organised by practitioners within a setting. The particular breakdown in this instance was troublesome for the digital plumbers working on installs as it was something that did not fit their understanding of how the system should behave in that context. Something which Orr (1996) noted as, a situation that both holds the interest of technicians and makes them feel uncomfortable.

Visibility within the WISP team’s exploration process was achieved in a number of ways as the team worked together to systematically foreground the relevant information to their area of experience and expertise. Firstly we see **accounts of experienced behaviour**, these are presented primarily by Tom as part of his role as Technical Operations Manager (over-seeing the digital plumbing team). Tom presented accounts of installations through descriptions of the steps taken by the engineer and the behaviour experienced from the device. The behaviour is reported by Tom as bothersome and something which did not fit with the technicians understanding of how things ‘should be’. While it does not compromise the performance of the system as such, it is unexpected. As Orr (1996) notes, it is these situations of unexplained and unexpected behaviour that often prompt technicians to share details with their team, thereby occasioning collaborative troubleshooting work. It is also worth noting here that, as a boundary object (Star, 2010) the internal monitoring system played a key role within this account sharing work. Tom used it initially to gain further visibility during the breakdown regarding the status of the device. It was also used to give further context to his accounts during the meeting through interaction and error logs stored within this cloud system. Finally we note that it was used by Luke (the Test and Verification Engineer) to observe the hidden behaviour of ‘wandering’ around the channels. This additional information was used to link the ‘join’ and ‘rejoin’ processes allowing the team to assess and address a seemingly related problem. The internal monitoring system, in this respect, serves to provide a glimpse of these relational aspects, although, much of the technical and operational work involved in the development and management of a solution of this nature remains invisible to the different perspectives. Hence the need to support it with focused knowledge sharing processes to further explore relational tensions between different object worlds (Bucciarelli, 1988) when breakdowns occur.

The second way in which we see the team systematically foreground situated knowledge is through **reports of expected behaviour**. Dean as the software developer with the relevant knowledge and expertise responds to Tom’s accounts with an explanation of the context that would cause this observed behaviour and the way in which the device is programmed to respond to this context. Dean’s explanations are thus produced to elaborate upon, and contextualise—and in doing so create a relation to Tom’s reports. The final stage of this process was for Tom to present information about the practical time challenges of the on-site engineer, highlighting the tensions between the device behaviour and how that device behaviour relates in situ to actual engineer practices.

The findings presented here demonstrate the ways in which the team traced **relations between experienced and expected behaviour** to find the underlying cause of the breakdown as connection difficulty. We also see how they further traced the relational aspects to identify a connected issue through similar behaviour. The result of this **systematic exploration work** was the surfacing of tensions between the goals of the engineer and that of ‘the device’. As the device itself struggles to connect to an unknown channel the engineer must wait, something which is incongruous with the efficiency goals of the engineer. Partly from an operational perspective in terms of business time costs, but also potentially as something that could impact the engineer customer relations if viewed as a lack of knowledge or skill (Orr, 1996). Furthermore, the ‘knowledge’ which the device is searching for is already known (or at least easily accessible) to the engineer. The tension is there because the engineer is on-site at the time, waiting around, which is something that was not the case with the ‘rejoin’ process. This process of identifying tensions thus generated a new focus, understanding and basis for the team to move forwards with the detailed design work. In summary the process of knowledge sharing in this way serves to define the notions of on-siteness and connectedness as infrastructural elements in tension with each other. This is somewhat similar to the defining work of fasting times described by Simonsen et al. (2020) as an early step in the design process. In our case, however, this is a much less formal process surfaced through behavioural accounts and resulting in a broad framing for the subsequent ‘fleshing out’ work by foregrounding these two core elements.

### Notions of on-siteness and connectedness as resources for design reasoning

In addition to identifying a relevant point of infrastructure to situate innovation, the process of exploring the trouble helps to frame the subsequent design work. Within the exploration work the team begin to develop the core notions of elemental states central to the design problem. Based on the collective knowledge that the shared goals and values are ‘establishing a connection’ and ‘efficiency of the operational elements’ (i.e. time engineer is required on-site), the team develop notions of connectedness and on-siteness. These are then drawn on as resources for design providing a framework for the team to anticipate and assess potential impacts as they **trace relations** to flesh out the solution. These are directly related to the tensions between the installed bases of the technological process for establishing a connection and the work process of the on-site engineer, surfaced during the first stage of the meeting. By drawing on opportunities and limitations of these installed bases (Star and Bowker, 2006) we see these notions of elemental state used to support design reasoning through continually foregrounding, thereby providing visibility over the tensions identified during the exploration of the installation troubles. We describe each of these notions in more detail in the following sections.

#### Connectedness as a representation of networked state

Connectedness in this case, is used by the team as a notion with 4 states. These are: not yet connected, reliably connected, unreliably connected, attempting to connect. Within the search process stage of the meeting the tensions raised as the cause of breakdown were in relation to the device attempting to connect. As the team worked up the finer details of the solution they considered specific use cases and drew on the notion of connectedness to consider questions or suggestions. For example, Tom questioned the feasibilty of using an RFID tag during installation by stating ‘we may not have that connection’ and relating it to similar functionality (i.e. the cleaner’s RFID tags), a concern which Dean dismissed as the owner’s tags are processed locally meaning a connection is not required. In this way Tom uses the notion of connectedness to further explore the feasibility of the solution by tracing it through similar functionality and comparing the connection state of the two examples. A further example of this can be seen in Fragment 3, where Dean suggested the connection is reliable at the point the wake-up channel needs configuring. In this example relational tracing was used to consider similar functionality as the team tried to anticipate and prevent similar breakdowns. Our final example is in Fragment 4 as John suggests the connection may be problematic and therefore not reliable if a whole site channel change is required. Again the connection state of the device was used to anticipate and evaluate the feasibility of suggested functionality changes. These notions of connectedness were used in this way throughout the design process as a resource for design reasoning to allow the team to evaluate the conditions and potential impact of the installed base.

#### On-siteness as a representation of operational priority

While the notion of connectedness can be thought of as physical in nature, the notion of on-siteness is more complex. While it has 3 states (on-site installation and maintenance, off-site maintenance and no engineer required) these are not as clearly defined as the notions of connection. They incorporate goals and values, for example, a state of engineer already on-site alludes to requirements of minimising time, utilising knowledge, having the correct tools. The state off-site is less restricted by time but is reliant on visibility and control through the cloud based product and/or monitoring systems. While the on-site state is used as a resource for design reasoning in the same way as off-site and no engineer required, it is the primary state in relation to the breakdown and innovation being discussed here. For that reason it also serves to maintain focus on ensuring adequate support for the ‘digital plumber’ is achieved within the troubleshooting and design session. With the initial breakdown occurring whilst an engineer was on-site Tom explicitly provided additional information relating to this state within his accounts. The states of off-site and no engineer required are more aligned to general business goals and values shared as part of membership in the project team. In this way it is quite different to the state of connectedness as it is directly linked to stakeholder values.

With its roots deep in business goals and values, **on-siteness often overrides connectedness** in its weighting for design reasoning. For example, Fragment 4 shows Tom suggesting that if a site wide channel change is needed he would like to be able to do it remotely without an engineer having to visit each node. John points out that this may be problematic as the channel is likely to be being changed due to unreliability. While it is acknowledged that the connection state is ‘unreliable’ the goal of the ‘no engineer required’ state is considered something worth attempting. While they may mitigate this risk by having an engineer nearby and making the customer aware when the change is made, it is still deemed beneficial to attempt it remotely. Another interesting example of the notion of on-siteness being used by the team was in Fragment 3 where Dean suggested that the wake-up channel configuration could be done either automatically, without the need for an engineer, or manually ‘off-site’ via the cloud system, thereby reducing the need for an engineer visit. This suggestion was subsequently dismissed by Simon the R&D manager based on the premise that an engineer would already be on-site as it is configured during the installation process. Once an engineer is on-site they will have limited time to wait for an automated process, particularly when they have the tools and knowledge already in place to set it manually at the same time as the operational channel.

These operational notions we have uncovered within WISP’s infrastructuring work, builds on previous research in this area, providing further insights into the ways in which background elements of infrastructures can be made visible and drawn upon as a resource to guide and support design work as it happens. In particular these notions bear some resemblance to the way in which Simonsen et al. (2020) describe “*characterizations, categorisations and considerations of infrastructural relevance and consequence*”. As such these notions of elemental state further illustrate infrastructural inversion used in a generative way to support design. However, in our case it is the troubleshooting activities of **reporting experienced and expected behaviours in tension at the point of breakdown** that initially bring these notions to the fore, as opposed to the intentional design work described by Simonsen et al. (2020). In this way we build on previous descriptions of infrastructural inversion (Bowker, 1994) through the identification and demonstration of the use of **notions of elemental states** to provide purchase to stakeholders as they collaboratively move through design reasoning work towards an integrative solution.

This approach to infrastructural inversion served to provide the WISP team a framework with which to remain focused on the point of breakdown, while at the same time predicting other potentially related breakdowns as a result of their planned changes to both the technology and the on-site work processes. By building on these previous examples of infrastructural inversion, our contribution here is to uncover *how* practitioners can use this to gain purchase from tensions within the installed base and navigate the key challenge of invisible-in-use but visible-for-design, noted as central in both infrastructuring (Neumann and Star, 1996) and ubiquitous computing (Tolmie et al. 2002) literature.

### Expanding on the work of the digital plumber

In this paper we have presented accounts of a project team addressing and resolving trouble experienced by professional ‘digital plumbers’ (Tolmie et al. 2010; Castelli et al. 2022). Our study shows the development of a new installation process that incorporates an RFID tag to be used by the digital plumber to set the channel of the device. The key fob is both a new physical tool for the digital plumber *and* part of the information layer of the infrastructure itself. Its purpose is to transfer knowledge in the form of data from the on-site engineer to the software on the HUB, essentially providing a link between the maintenance work process and technological layers of the Alert solution. However, although the fob was central to the design change being made, curiously it did *not* feature as part of the design process and was not treated as an object of design in and of itself. So, the RFID tag as a solution draws on built-in capabilities of the devices, effectively embodying standards that already exist within the current solution. It is therefore the surrounding work of digital plumbing and the associated software processes that are the subject of change.

Our findings demonstrate how Tom, the Technical Operations Manager, held a key role within the entire redesign process, from initially raising the trouble experienced during the field trials right through to executing and communicating the change to the engineering team. As part of this role of Digital Plumbing Representative, Tom had a number of responsibilities to fulfill. Firstly, he needed to raise the issue as worthy of a meeting, then during the meeting, further escalate this as something which needed a resolution. This was achieved through providing accounts of a breakdown. In a way that resonates strongly with observations discussed by Orr (1996), Tom provided reports of specific installs for which he had personally been involved in which he had encountered trouble, he supported these with logs from the internal monitoring system and included enough information for Dean to identify whether this was a software bug or expected behaviour. Another responsibility held by Tom was that of knowledge sharing the constraints of the digital plumber’s role and the current processes and procedures relevant to the task at hand. Further to this he identified and presented a solution based on an understanding of the channel knowledge accessible to the on-site engineer and the technical problem as it had been explained to him. Finally, Tom fulfilled the role of redesigning the installation w ork process to coincide with the planned technology changes.

Through the unpacking of Tom’s role in this way we are able to build on the four major areas of digital plumbers work identified by Tolmie et al. (2010). These are preparatory work, assembly of tools and parts, management of contingency and coordination and awareness. While some of the design related duties we have identified could be seen as linked to the management of contingencies, we suggest that due to the size of the responsibility involved in this area of work an additional area of supporting the facilitation of change is added. In a similar vein to Pipek and Wulf (2009), we suggest open communication channels between the digital plumbing team and internal development team is essential to support user-centered design through the communication of infrastructural visibilities and the assessment of stakeholder values. We suggest it is essential that where modifications are being made to the installation or maintenance elements of the product there is a digital plumbing representative at the table. This is particularly necessary in cases such as this where the project relates to a fully managed commercial IoT solution to ensure consistency of service. Thus we also build on more recent reports of the digital plumbing role within the professional context (Castelli et al. 2022) by highlighting further challenges faced by professional digital plumbers within industry settings. In this context we consider the digital plumber as a kind of super-user with facilitating change as a key part of the role. In the way that the WISP team set up field trials within Project Rock the field engineers were represented, by Tom, as a group that was expected to assess and feedback on the findings of the trial in relation to their work. This formalised field trial approach supported a detailed collaborative investigation when trouble was encountered, resulting in the conditions for process and technology changes to occur in tandem.

### Implications and future work

#### Supporting other users

IoT is a varied industry with many different products, services, markets and solutions. Within this type of B2B managed solution, there are at least three different stakeholder groups: in-house engineers, the work-force at the deployment site (i.e. the customer business), and the end users (in our case occupants of the dwellings) that interact directly with the devices. While this paper focuses on a troubleshooting meeting incorporating a design session with the in-house engineers—notably an expert user group—sessions with external customers are likely to be very different as goals and values are not aligned under the same business structure. The work infrastructure of the customer site is integrated through the solution so may share values in that respect, but is separate from an organisation perspective.

Within our case the team were not attempting to ‘do design’ in a formal sense. This was an impromptu session that led on directly from troubleshooting activities in the first half of the meeting. Thus there was not a design plan or structured session and there were no ‘designers’ leading or facilitating the session. Instead the role of designer belonged to everyone and no-one. Each member was aware of their own and others local accountabilities and drew on the collective knowledge and notions built up throughout the meeting and through their membership in various object worlds (Bucciarelli, 1988) within the business, the project and the meeting to work up supporting design changes within their remit to ensure the success of the new RFID channel setting solution. This raises questions about how other user groups could be incorporated into a this type of design work and ways in which the work would unfold differently if it was ‘facilitated’ as a design session. As Orr (1996) has clearly outlined the technicians skill set is inclined towards troubleshooting and sharing details of encountered problems. We therefore suggest a similar situation arising from an infrastructural breakdown incorporating different stakeholder or user groups would potentially require more facilitation and support as the specific analytical skill set of the digital plumber in the study of this paper clearly played a key role in that respect. We suggest future research is necessary to unpack these different scenarios.

As HCI and CSCW extends concerns into the longer-term aspects of Ubicomp design and management, deeper understanding around the situated work of practitioners within commercially managed IoT solutions becomes a valuable resource to enrich our understanding of concepts like the ‘digital plumber’ as well as commercial infrastructures ‘in the wild’. Through our ethnographic study we shed new light on the collaborative accomplishment of troubleshooting difficulties and designing changes to an established installation and maintenance processes. Our contribution is twofold. Firstly we contribute to the infrastructuring discourse through presenting observations of infrastructural inversion being used to generate resources which frame and give purchase to the subsequent design work. Thus extending previous knowledge through insights into how infrastructuring is materially practiced from a troubleshooting session resulting from an infrastructural breakdown within a fully deployed well established IoT system. We do this through responding to ideas about infrastructuring with respect to our findings to unpack the ways in which a team producing and managing IoT solutions explore and resolve trouble raised during field trials and provide a **demonstration of the use of infrastructural inversion to support design reasoning**. Finally, through presenting a practice-oriented study relating to the service based elements of delivering IoT we contribute to the movement towards a holistic view of Industry 4.0 (Ludwig et al. 2018). In doing so, we extend the view of the digital plumber, building on previous works considering the undertaking of this work by researchers (Tolmie et al. 2010), householders (Grinter et al. 2009) and third party engineers (Castelli et al. 2022), to a professional practice of in-house field engineers as part of an ongoing managed service. As such, we present **additional responsibilities relating to the work of digital plumbing** as also including work of **providing accounts of behaviour** relating to infrastructural breakdowns and where change is deemed necessary, **partaking in design work** to redesign processes and technology solutions.

## Conclusion

In this paper we have reported on the ways in which a commercial project team navigated the challenges of addressing a trouble raised during a field trial of an updated IoT alarm system. We note how the digital plumber raised the issues he was experiencing during installations and then facilitated an investigation exploring the trouble and working towards a solution. Our findings show the ways in which the team used techniques of Infrastructural Inversion to support their search for accountability and to uncover tensions leading to a resolution. Our contribution here is in the **demonstration of the use of notions of elemental states**, generated from the initial process of exploring the trouble and carried forward as a resource to **maintain visibility over infrastructural elements** to support and guide the design work during the ‘fleshing out’ phase of the work. This builds on previous demonstrations of infrastructuring in practice drawing on the concept of infrastructural inversion (Bowker, 1994) as a generative tool for design work (Simonsen et al. 2020). Our second contribution is the **identification of additional responsibilities within the role of the professional digital plumber** which build on previous descriptions (Tolmie et al. 2010; Castelli et al. 2022). We identify the additional responsibilities of reporting of infrastructural breakdowns alongside user-led innovation and facilitation of change.

## Footnotes

1 It is important to state here that the notion of digital plumbing used in this paper, in line with current literature, refers to the onsite installation and maintenance of hardware.

2 While adding bidirectional communication was the main purpose of Project Rock, inevitably, as with many commercial modification projects, additional bug fixing and optimisation tasks were also included in the project’s code version releases

3 Note here some interesting frictions with ubicomp’s discussions of ‘invisibility’.

4 It is important to note that when we talk about connectedness in this sense we are talking about the physical state of the networked device and how that state influences the relationship and knowledge balance between the engineer and the device (as a non-human actant) within the task of installation. This is more limited than the way the term is often used in the infrastructuring discourse referring to an infrastructures reach or scope beyond a single event or practice
